# Insights into the Recruitment of Class IIa Histone Deacetylases (HDACs) to the SMRT/NCoR Transcriptional Repression Complex*

**DOI:** 10.1074/jbc.M115.661058

**Published:** 2015-06-08

**Authors:** Gregg M. Hudson, Peter J. Watson, Louise Fairall, Andrew G. Jamieson, John W. R. Schwabe

**Affiliations:** From the ‡ Department of Biochemistry, Henry Wellcome Laboratories of Structural Biology, University of Leicester, Leicester LE1 9HN and; the § Department of Chemistry, University of Leicester, Leicester LE1 7RH, United Kingdom

**Keywords:** epigenetics, histone acetylation, histone deacetylase 4 (HDAC4), protein-protein interaction, transcription corepressor

## Abstract

Class IIa histone deacetylases repress transcription of target genes. However, their mechanism of action is poorly understood because they exhibit very low levels of deacetylase activity. The class IIa HDACs are associated with the SMRT/NCoR repression complexes and this may, at least in part, account for their repressive activity. However, the molecular mechanism of recruitment to co-repressor proteins has yet to be established. Here we show that a repeated peptide motif present in both SMRT and NCoR is sufficient to mediate specific interaction, with micromolar affinity, with all the class IIa HDACs (HDACs 4, 5, 7, and 9). Mutations in the consensus motif abrogate binding. Mutational analysis of HDAC4 suggests that the peptide interacts in the vicinity of the active site of the enzyme and requires the “closed” conformation of the zinc-binding loop on the surface of the enzyme. Together these findings represent the first insights into the molecular mechanism of recruitment of class IIa HDACs to the SMRT/NCoR repression complexes.

## Teaser

**Background:** Class IIa histone deacetylases (HDACs) repress transcription through association with the SMRT/NCOR co-repressor complex.

**Results:** A repeated peptide motif mediates recruitment of class IIa HDACs to the co-repressor proteins interacting adjacent to the active site.

**Conclusion:** Class IIa HDACs are recruited to co-repressors by a simple repeated peptide motif.

**Significance:** First insights into the assembly of Class IIa HDACs with repression complexes.

## Introduction

The post-translational acetylation of lysine residues plays an important role in regulating the activity of many proteins (1). Levels of acetylation are controlled through the opposing action of acetyltransferase and deacetylase enzymes. These enzymes were first characterized as a consequence of their role in regulating gene expression through controlling the acetylation of lysine residues in the tails of histone proteins. In humans there are 18 deacetylase enzymes (often called histone deacetylases or HDACs),^2^ which can be grouped into four classes on the basis of sequence conservation (reviewed in Ref. 2).

The class I HDACs 1–3 are nuclear proteins that play roles in regulating multiple genomic activities including transcriptional repression, DNA repair, and replication (reviewed in Ref. 3). Importantly, they require assembly into cognate co-repressor complexes for full activity and genome targeting (4–10). The structural basis for the interaction of HDACs 1 and 3 with their cognate corepressors MTA1 and SMRT/NCoR has recently been established. These structures show that SANT domains in the cognate co-repressor proteins play a key role in activating the HDACs through mediating interaction with inositol phosphates, which are essential for full deacetylase activity (11, 12).

Class IIa HDACs are also predominantly nuclear proteins, but, unlike the class I HDACs that consist of a single catalytic domain, the class IIa HDACs contain an N-terminal extension of ∼600 residues. This N-terminal domain provides a platform for several protein-protein interactions and post-translational modifications (13–15). Importantly, although class IIa HDACs also act to repress transcription their mechanism of action appears to be distinct from that of the class I HDACs. In particular, their transcriptional repression activity may, at least in part, be independent of the catalytic domain of the deacetylase (16, 17). Indeed, the catalytic domain itself has a very weak deacetylase activity, which correlates with a histidine residue, unique to class IIa HDACs. In the class I enzymes, the equivalent residue is a tyrosine and mutation of the histidine to a tyrosine greatly increases the activity of the class IIa enzymes (18–20).

Importantly, like the class I enzymes, the class IIa HDACs 4, 5, and 7 have been shown to interact through their catalytic domain, with the co-repressors SMRT and NCoR. However, this interaction does not involve the conserved SANT domain of the corepressor and has instead been mapped to a region (repression domain 3 or RD3) that is predicted to be intrinsically disordered (21–23).

To date we have only limited understanding of the nature of the interaction between class IIa HDACs and the SMRT/NCOR co-repressors. Here we show that a conserved and repeated sequence motif within the SMRT and NCoR corepressors directly mediates interaction with all four class IIa HDACs. We also show that mutations around the active site of HDAC4, as well as the binding of HDAC inhibitors modulate the interaction with co-repressors.

## Experimental Procedures

### 

#### 

##### Preparation of Recombinant HDAC4

Histone deacetylase 4 was expressed with a hexa-histidine tag, S-tag, and tobacco etch virus protease cleavage site at the N terminus using a pET30 expression vector in the Rosetta strain of *Escherichia coli* (Invitrogen). Cells were grown in 2YT medium supplemented with 34 μg/ml of chloramphenicol and 50 μg/ml of kanamycin. Cells were grown at 37 ºC to an *A*_600_ 0.8–1.0 before being supplemented with 50 μm zinc acetate for 30 min prior to induction with 40 μm isopropyl β-d-thiogalactopyranoside then grown for 16 h at 18 ºC. Cells were harvested by centrifugation at 5000 × *g* and resuspended in lysis buffer (25 mm HEPES, pH 7.5, 200 mm potassium chloride, 30% glycerol, 1 mm dithiothreitol, and a complete EDTA-free protease inhibitor tablet (Roche Applied Sciences)). 20 mm magnesium chloride and 100 μl of 10 mg/ml of DNase I (Sigma) was added to the suspended cells prior to lysis using a C3-EmulsiFlex (Avastin Inc.) at 22,500 p.s.i. Lysates were clarified by ultracentrifugation (110,000 × *g*, 25 min, 4 ºC) before binding to pre-equilibrated Ni-NTA resin (Qiagen) for 30 min. HDAC4 was diluted with an equal volume of dilution buffer (25 mm HEPES, pH 7.5, 200 mm potassium chloride, and 1 mm dithiothreitol) prior to binding to resin. The resin was extensively washed with wash buffer (25 mm HEPES, pH 7.5, 200 mm potassium chloride, 5% glycerol, 1 mm dithiothreitol, and 20 mm imidazole, pH 8.0). HDAC4 was eluted with 6 volumes of elution buffer (25 mm HEPES, pH 7.5, 200 mm potassium chloride, 5% glycerol, 1 mm dithiothreitol, and 150 mm imidazole, pH 8.0). Protein was dialyzed at 4 ºC overnight (25 mm HEPES, pH 7.5, 20 mm potassium chloride, 10% glycerol, and 1 mm dithiothreitol). The protein was further purified on a HiTrapQ ion exchange column (GE Healthcare) at 4 ºC against a salt gradient (25 mm HEPES, pH 7.5, 50–500 mm potassium chloride, 1 mm dithiothreitol). The protein was cleaved with tobacco etch virus protease at 4 ºC overnight before a final purification using a Superdex S200 column at 4 ºC (25 mm HEPES, pH 7.5, 200 mm potassium chloride, 1 mm dithiothreitol). HDAC4 was concentrated using Amicon centrifugal concentrators before use.

##### Preparation of Recombinant HDAC7

Histone deacetylase 7 was prepared in the same manner to HDAC4, however, the high speed supernatant of HDAC7 was not diluted prior to incubation with Ni-NTA resin and the following buffers were used. Lysis buffer was 1× PBS, pH 7.4, 500 mm sodium chloride, 5% glycerol, 0.1% CHAPS, 1 mm dithiothreitol, 25 μg/ml of 4-(2-aminoethyl)benzenesulfonyl fluoride and a complete EDTA-free protease inhibitor tablet. Wash buffer was 20 mm Tris, pH 8.0, 250 mm sodium chloride, 0.1% CHAPS, 1 mm dithiothreitol, and 20 mm imidazole, pH 8.0. Elution buffer was 20 mm Tris, pH 8.0, 250 mm sodium chloride, 0.1% CHAPS, 1 mm dithiothreitol, and 250 mm imidazole pH 8.0. Dialysis buffer was 20 mm Tris, pH 8.0, 150 mm sodium chloride, 5% glycerol, and 1 mm dithiothreitol. Ion exchange buffer was 20 mm Tris, pH 8.0, 50–500 mm sodium chloride. Gel filtration buffer was 20 mm Tris, pH 8.0, 100 mm sodium chloride.

##### Preparation of Recombinant HDACs 5 and 9

Histone deacetylases 5 and 9 were prepared in the same manner to HDAC4, however, the high speed supernatants were not diluted prior to incubation with Ni-NTA resin and the following buffers were used. Lysis buffer was 45 mm Tris, pH 8.0, 120 mm sodium chloride, 2.5 mm potassium chloride, 15% glycerol, 1 mm dithiothreitol and a complete EDTA-free protease inhibitor tablet. Wash buffer was 45 mm Tris, pH 8.0, 120 mm sodium chloride, 2.5 mm potassium chloride, 10% glycerol, 1 mm dithiothreitol, and 20 mm imidazole, pH 8.0. Elution buffer was 45 mm Tris, pH 8.0, 120 mm sodium chloride, 2.5 mm potassium chloride, 10% glycerol, 1 mm dithiothreitol, and 200 mm imidazole pH 8.0. Dialysis buffer was 45 mm Tris, pH 8.0, 50 mm sodium chloride, 2.5 mm potassium chloride, 10% glycerol and 1 mm dithiothreitol. Ion exchange buffer was 45 mm Tris, pH 8.0, 50–500 mm sodium chloride, 2.5 mm potassium chloride, 1 mm dithiothreitol. Gel filtration buffer was 45 mm Tris, pH 8.0, 120 mm sodium chloride, 2.5 mm potassium chloride, 1 mm dithiothreitol.

##### Peptide Production

Wild type peptide was obtained from Biomatik. Mutant peptides were synthesized in house using a CEM microwave peptide synthesizer. Peptides for use in fluorescence anisotropy assays were synthesized with an extra N-terminal cysteine.

##### Fluorescence Anisotropy

Peptides were coupled to BODIPY-TMR by incubation in 18 mΩ water at a 5:1 molar ratio for 2 h at room temperature with continual stirring. Following incubation, buffer was exchanged into 1× PBS, 0.5 mm Tris(2-carboxyethyl)phosphine using a PD-10 column (GE Healthcare). Labeled peptides were concentrated using an Amicon centrifugal concentrator. Immediately prior to the fluorescence anisotropy assays the HDACs were concentrated and buffer exchanged into 1× PBS, 0.5 mm Tris(2-carboxyethyl)-phosphine and the protein concentration was determined by *A*_280_ in 6 m guanidine HCl. Protein was dispensed into 96-well black plates prior to a 2-fold serial dilution in reaction buffer (1× PBS, 0.01% Triton X-100, 0.1 mg/ml of BSA). Inhibitors were preincubated with HDACs for 30 min at a 2:1 ratio before serial dilution. Labeled peptide was added to a final concentration of 1 μm in a 50-μl reaction volume. Measurements were recorded using an excitation wavelength of 531 nm and emission wavelength of 595 nm. Data were analyzed using GraphPad Prism, assuming non-linear fit with one-site binding to fulfill the equation *y* = *B*_max_ × *x*/(*K_d_* + *x*).

##### Histone Deacetylase Activity Assays

A fluorescent HDAC assay (Active Motif) was used to determine HDAC activity. Protein was diluted to the desired concentration in assay buffer (20 mm Tris, pH 7.5, 0.25 mm EDTA, 250 mm sodium chloride) before incubation for 90 min at 37 ºC with 100 μm BOC-acetyl-lysine substrate. The assay was terminated by incubation with developer solution (2 μm trichostatin A, 10 μg/μl of trypsin, 50 mm Tris, pH 7.5, 100 mm sodium chloride) for 10 min at 25 ºC. Measurements were recorded using an excitation wavelength of 355 nm and an emission wavelength of 460 nm.

## Results

### 

#### 

##### Identification of a Consensus GSI Motif in RD3 of SMRT/NCoR

**FIGURE 1.**
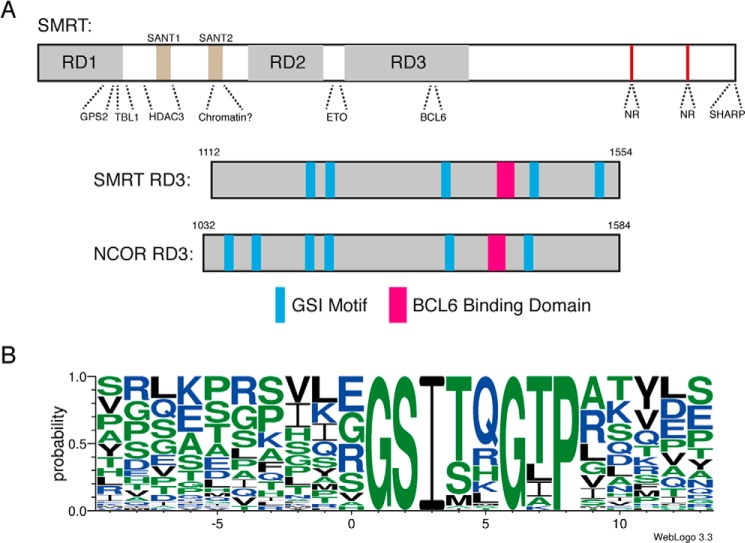
**A repeated sequence motif within the class IIa HDAC binding region of SMRT/NCOR co-repressors.** *A,* domain arrangement of SMRT indicating regions known to interact with partner proteins and schematic of RD3 of SMRT/NCoR showing the location of identified GSI repeats and the BCL6 binding domain with a similar arrangement and spacing. *B,* Weblogo plot of RD3 GSI motifs from SMRT and NCoR of 6 species demonstrates a well conserved 8 amino acid residue motif. *NR,* nuclear receptor binding region.

The so-called repression domain 3 (RD3) of SMRT and NCoR has previously been shown to interact both with the repressive transcription factor BCL6 and with the class IIa HDACs (16, 21–23). Sequence analysis of the RD3 regions of SMRT and NCoR indicates that these regions are intrinsically unstructured. Strikingly, within the RD3 domain, we observed a repeated 8-amino acid motif with a consensus sequence G-S-I-t/s-q-G-t-P (capitals indicate absolute conservation). In SMRT and NCoR there are 5 and 6 GSI motifs, respectively, with an apparent core conserved region consisting of 4 motifs flanking the BCL6 binding domain (Fig. 1*A*). Alignment of >70 GSI motifs and the flanking regions from multiple SMRT/NCoR homologues suggests that the sequence conservation is restricted to the core eight amino acids (Fig. 1*B*) suggesting an important functional role for this motif. Interestingly a scan prosite search using GSI(S/T)*X*G*X*P as a search pattern indicates that this motif is not found in any other proteins in the human proteome. We hypothesized therefore that these GSI motifs might play a role in mediating interaction of co-repressor proteins with the class IIa HDACs.

##### Interaction of GSI Motifs with Class IIa HDACs

**FIGURE 2.**
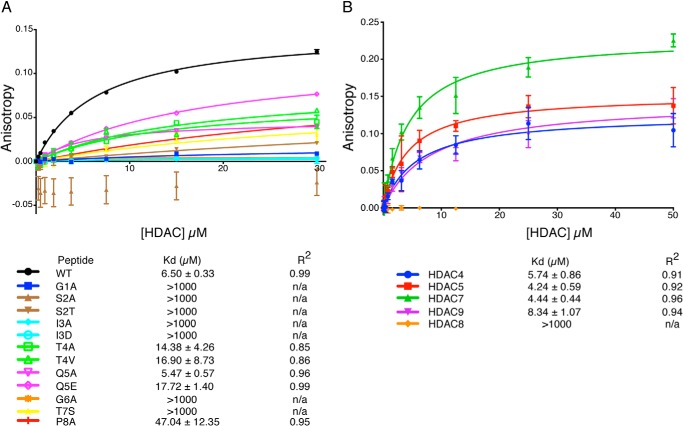
**Investigating the interaction between class IIa HDACs and a GSI motif peptide (CPRPLKEGSITQGTPLKYDTG).** *A,* a peptide representative of a wild type GSI motif and 12 mutant peptides were fluorescently labeled for use in fluorescence anisotropy to define and characterize the interaction between the peptide and HDAC4. *B,* fluorescence anisotropy was performed using the wild type peptide with the catalytic domains of class IIa HDACs HDAC4, HDAC5, HDAC7, and HDAC9 and the class I HDAC8.

To determine whether GSI motifs do indeed mediate interaction with class IIa HDACs we synthesized a peptide corresponding to amino acids 1450–1469 from the SMRT, which contains a perfect GSITQGTP consensus motif and an amino-terminal cysteine. The cysteine was chemically coupled to a BODIPY fluorophore. Fluorescence anisotropy measurements using the labeled peptide and bacterially expressed HDAC4 indicated a saturable interaction with a low micromolar dissociation constant similar to that of other protein-protein interactions involving the co-repressor complexes (24–26) (Fig. 2*A*).

To explore the sequence dependence of the interaction with HDAC4 we synthesized 12 peptides in which the GSITQGTP sequence was systematically altered (Fig. 2*A*). Strikingly, even conservative mutations in Gly-1, Ser-2, Ile-3, Gly-6, and Thr-7 essentially completely abolished interaction with HDAC4 suggesting that these side chains are involved in stereospecific interactions with the catalytic domain of the HDAC. Mutations to Thr-4, Gln-5, and Pro-8 did not reduce binding to the same extent suggesting that these residues are less important for the interaction. The lower conservation of the amino acid in position 5 fits well with this finding. In contrast, nearly 90% of the GSI motifs have a threonine or serine at position 4. This suggests that the hydroxyl group shared by these residues is important in some way, even though it is not required for interaction with HDAC4.

To determine whether other class IIa HDACs can also be recruited to the SMRT/NCoR corepressors through the GSI motif, we purified the recombinant catalytic domains of HDACs 4, 5, 7, and 9, along with full-length HDAC8 as an intrinsically active class I HDAC control (27). Fluorescence anisotropy assays clearly show that all four class IIa HDACs can interact with the GSI motif peptide with similar binding affinities (Fig. 2*B*). The class I HDAC8 shows no interaction with the peptide supporting the hypothesis that the GSI motif interaction is specific to class IIa HDACs.

##### Insights into the Interaction of GSI Peptides with HDAC4

To understand the nature of the interaction of GSI motifs with HDAC4 we set up many crystallization trials of the complex, but were ultimately unsuccessful in obtaining diffraction quality crystals. However, it has previously been reported that the presence of an HDAC inhibitor may influence the interaction between HDAC4 and the co-repressors complexes suggesting that there may be cross-talk between the active site and the co-repressor interaction interface (19, 20, 28).

**FIGURE 3.**
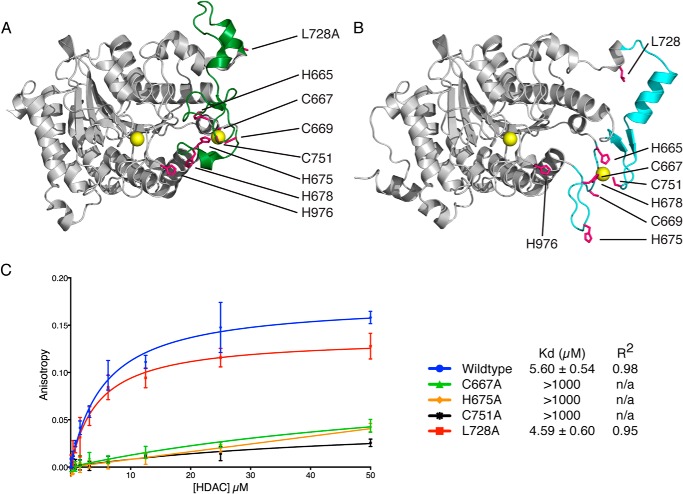
**Comparison of the closed (*A*) and open (*B*) loop conformations of HDAC4.** Views of a wild type HDAC4 structure (Protein Data Bank code 2vqj) with an open loop conformation (*cyan*) and an L728A mutant structure (Protein Data Bank code 4cby), which demonstrates a closed loop conformation (*green*). Side chains of the residues discussed in the text are highlighted in *magenta*. Zinc atoms are colored *yellow. C,* fluorescence anisotropy binding experiments of GSI peptide to wild type HDAC4cd, L728A mutant, which is reported to stabilize the class IIa loop, and three mutants of the zinc chelating residues, C667A, H675A, and C751A.

Importantly, structural studies of HDAC4 have shown that two structured loops (amino acids 669–679 and 729–765) can adopt very different configurations in different structures (Fig. 3, *A* and *B*) (20). In the so-called “closed conformation” (seen in the structure of the H976Y gain-of-function mutation (20)) the two loops collaborate to form a tetrahedral zinc-binding site with three cysteines (Cys-667, Cys-669, and Cys-751) and one histidine (His-675). It has been suggested that inhibitor binding influences the conformation of these loops and favors an “open conformation” in which two of the zinc ligands are substituted (Cys-669 and His-675 substituted by His-678 and His-665, respectively (20)). We hypothesized that the co-repressor interaction surface may involve these surface loops, which are in proximity to the active site of the enzyme.

To test this we mutated 3 of the ligands for the zinc in the closed conformation (Cys-667, His-675, and Cys-751) (Fig. 3*A*). Strikingly we found that all three mutations abolished the interaction of HDAC4 with the GSI peptide strongly suggesting that the corepressor interface lies in the vicinity of these loops and requires the closed conformation of the surface loops (Fig. 3*C*). Interestingly the mutation of leucine 728 to alanine has also been shown to favor the closed conformation of the loops (29). We show that this mutation binds GSI peptides with the same affinity as the wild type enzyme.

**FIGURE 4.**
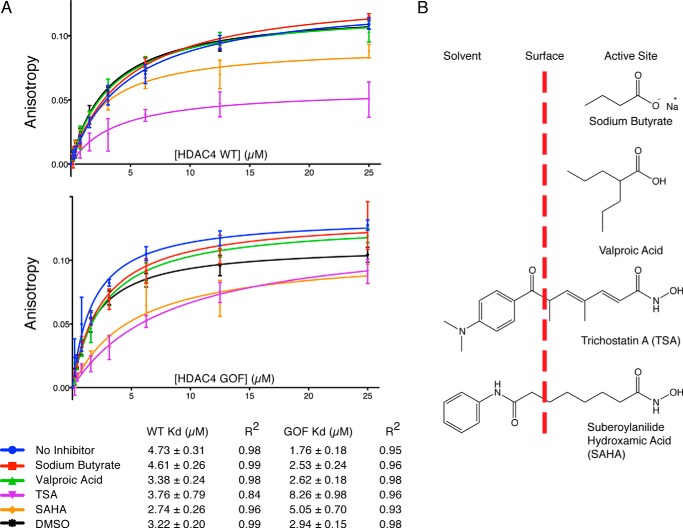
**Investigating the effect of inhibitors on the HDAC4-GSI interaction.** *A,* fluorescence anisotropy of wild type (*WT*) and the H976Y gain-of-function (*GOF*) HDAC4 catalytic domain with 2-fold molar excess of different HDAC inhibitors. *B,* schematic representation of how the different HDAC inhibitors occupy the active site.

To explore further whether there is communication between the GSI binding surface and the active site, we tested whether different HDAC inhibitors influenced the binding affinity of the GSI peptides to both wild type and H976Y gain-of-function HDAC4. Both proteins were incubated with a 2-fold molar excess of four HDAC inhibitors, suberoylanilide hydroxamic acid, trichostatin A, valproic acid, and sodium butyrate (Fig. 4*A*). Interestingly the gain-of-function mutant interacted with significantly higher affinity than the wild type supporting the hypothesis that GSI peptide binding is favored by the closed conformation of the enzyme. The four inhibitors did not greatly affect the affinity of GSI peptide binding to the wild type enzyme although the inhibitors that are large enough to protrude from the active site pocket (Fig. 4*B*), trichostatin A and suberoylanilide hydroxamic acid, gave a lower maximum polarization suggesting that the character of the inhibitor can directly or indirectly influence the mobility of the fluorophore attached to the GSI peptide. These larger inhibitors bound to the gain-of-function mutant result in significantly decreased affinity for the GSI peptide. Again this suggests cross-talk between the active site of HDAC4 and the co-repressor interaction surface. This raises the interesting possibility that co-repressor binding might play a role in activating the class IIa HDACs.

##### Corepressor Interactions Do Not Appear to Enhance the Deacetylase Activity of Class IIa HDACs

**FIGURE 5.**
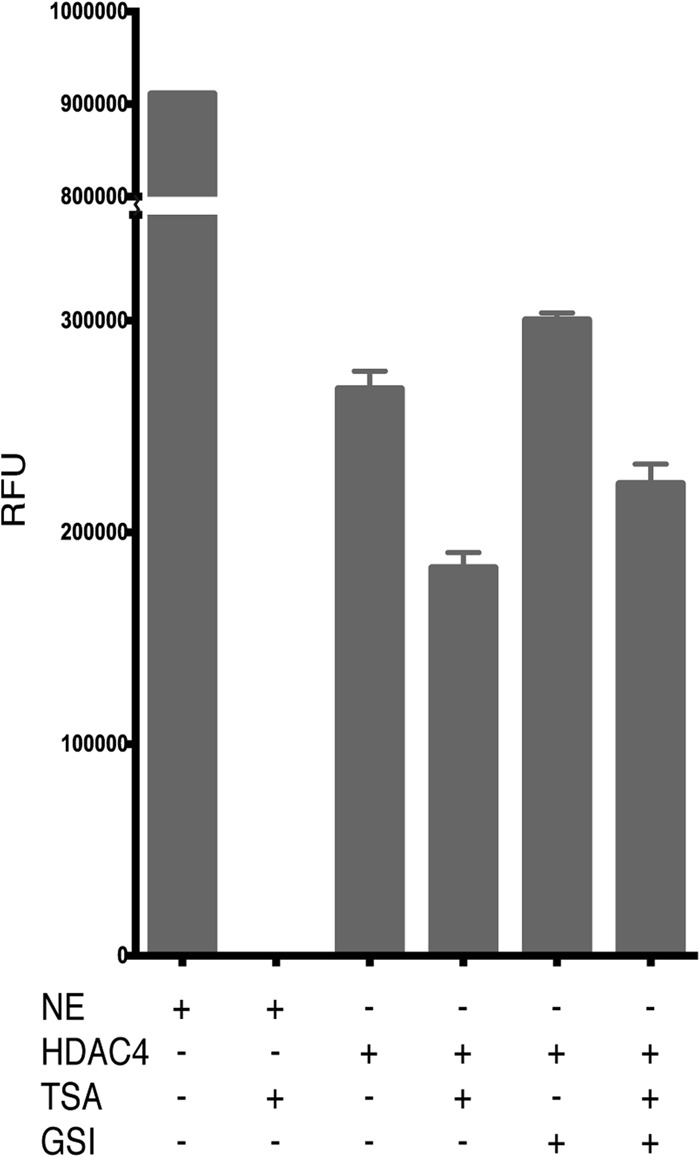
**Investigating the effect of co-repressor interaction on class IIa HDAC activity.** To determine whether interaction with co-repressor affects the deacetylation potential of the class IIa HDACs, an assay was performed using 5 μm HDAC4 catalytic domain in the presence and absence of GSI peptide (20:1) and inhibitor (2:1) compared with 5 μg of HeLa nuclear extract. *NE*, nuclear extract; *WT*, wild type HDAC4; *GSI*, GSI motif peptide; *TSA*, HDAC inhibitor trichostatin A.

The class I HDACs 1–3 are reliant upon their interaction with cognate co-repressor complexes to attain maximum activity. Because class IIa HDACs are generally thought to be inactive enzymes, we sought to investigate whether interaction with the co-repressor GSI motif might activate these enzymes in an analogous fashion to the class I HDACs. HDAC activity assays were used to determine whether the presence of an excess of the GSI motif peptide could enhance the activity of 5 μm HDAC4. Importantly, the presence of the GSI peptide appeared to have no effect on the lysine deacetylase activity of the HDAC4 catalytic domain (Fig. 5).

## Discussion

The molecular functioning of the vertebrate class IIa HDACs remains an enigma. They have a catalytic domain conserved back to yeast, which is homologous to the better understood class I HDACs. However, in the vertebrate class IIa enzymes a key amino acid at the active site is a histidine rather than the tyrosine that is essential for potent deacetylase activity (19). Thus these enzymes have a very low intrinsic lysine deacetylase activity. However, the active site is otherwise complete because mutagenesis of the histidine to a tyrosine restores a high level of deacetylase activity (19, 20). There are three possible interpretations: (i) the class IIa HDACs are not enzymatically active; (ii) they are activated by some unknown mechanism; or (iii) they have a different, but as yet unknown, enzymatic activity.

The concept that these enzymes might be activated in some way is particularly attractive because the class I HDACs 1–3 are known to be activated through assembly into co-repressor complexes and this activation requires the presence of inositol phosphates bound at the HDAC:corepressor interface (11, 12). If a similar scenario were to be true for the class IIa HDACs then it becomes important to understand how these are assembled into corepressor complexes.

In this study we have explored the recruitment of the class IIa HDACs to the SMRT/NCoR corepressor proteins. We show that class IIa HDACs interact specifically with RD3 of the SMRT/NCOR co-repressor complexes through an 8-amino acid GSI motif of which there are multiple occurrences of in each co-repressor. Although a single copy of the GSI motif is sufficient for a measurable interaction, it seems likely that the multiple copies increase the local concentration of the motif so as to increase the avidity and hence the likelihood of recruitment. We would suggest that it is less likely that the repeated GSI motifs facilitate the recruitment of multiple class IIa HDACs.

Our mutagenesis studies suggest that the GSI motifs interact in the vicinity of the active site of the class IIa HDACs and require the class IIa specific loop to adopt the so-called “closed” configuration. There appears to be communication between the active site and the stability of this loop because the binding of larger HDAC inhibitors both disfavors corepressor binding and appear to favor the open (or disordered) configuration of this loop. The importance of the loop configuration for corepressor binding is supported by the finding that the histidine to tyrosine gain-of-function mutation both favors the closed configuration of the loop and binds the GSI peptides more tightly.

It is unclear as to whether there are any native mechanisms that control a transition between the open and closed conformation of this loop, which may provide a regulatory process for the co-repressor interaction. However, it has been reported that reduction of one of the zinc chelating cysteines (Cys-669) by thioredoxin I alters the function of HDAC4 and prevents nuclear export (30). It is possible that this reduction promotes the loop to adopt the open conformation, which would also result in a loss of co-repressor interaction.

In contrast to the class I HDACs, the class IIa HDACs do not show any significant enhancement of lysine deacetylase activity when bound to the corepressor. Therefore, the role of class IIa HDACs in the SMRT/NCoR repression complex remains to be determined. It should not be forgotten that these enzymes all have an extensive amino-terminal domain that is likely to play a role in gene regulation.

Understanding the mechanism of action of the class IIa HDACs is important because multiple lines of evidence suggest that they are associated with human disease. Indeed, a mouse model of Huntington disease is alleviated when the levels of HDAC4 are reduced (31). Loss of HDAC5 impairs memory function (32). HDAC7 promotes apoptosis and c-Myc regulation in some leukemias and lymphomas, which may partly be due to the fact that it is a substrate for the extrinsic apoptosis protein caspase 8 (33, 34). Furthermore, a genome wide association study has identified a variant HDAC9 that is associated with large vessel ischemic stroke (35).

## Author Contributions

J. W. R. S. conceived and coordinated the study. G. M. H. performed the experiments. P. J. W. and L. F. guided G. M. H. in experimental execution and interpretation. A. G. J. supervised and guided the peptide synthesis. G. M. H. prepared the figures. G. M. H. and J. W. R. S. wrote the paper. All authors reviewed the results and approved the final version of the manuscript.

## Footnotes

2 The abbreviations used are:

HDAC: histone deacetylase
RD3: repression domain 3
Ni-NTA: nickel-nitrilotriacetic acid.
